# The magmatic evolution of South-East Crater (Mt. Etna) during the February–April 2021 sequence of lava fountains from a mineral chemistry perspective

**DOI:** 10.1007/s00445-023-01643-2

**Published:** 2023-04-26

**Authors:** Alessandro Musu, Rosa Anna Corsaro, Oliver Higgins, Corin Jorgenson, Maurizio Petrelli, Luca Caricchi

**Affiliations:** 1 Department of Earth Sciences, University of Geneva, Rue des Maraîchers 13, CH-1205 Geneva, Switzerland; 2 Istituto Nazionale di Geofisica e Vulcanologia, Osservatorio Etneo-Sezione di Catania, Piazza Roma 2, 95123 Catania, Italy; 3 Geology, School of Natural Sciences, Trinity College Dublin, Dublin, Ireland; 4 Department of Physics and Geology, University of Perugia, Piazza dell’Università, 1, 06123 Perugia, Italy

**Keywords:** Clinopyroxene, Hierarchical clustering, Mineral stratigraphy, Thermobarometry, Random forest machine learning

## Abstract

The South-East Crater (SEC) at Mt. Etna started a period of lava fountaining in December 2020, producing over 60 paroxysms until February 2022. The activity had an intense sequence from February 16 to April 1, 2021, totaling 17 paroxysmal events separated by repose times varying from 1 to 7 days. The eruptive sequence was extensively monitored, providing a unique opportunity to relate the chemistry and texture of the erupted products to eruption dynamics. We investigate the temporal evolution of the magmatic system through this eruptive sequence by quantifying variations in the composition and texture of clinopyroxene. Clinopyroxene major element transects across crystals from five representative lava fountains allow us to determine the relative proportions of deep versus shallow-stored magmas that fed these events. We use hierarchical clustering (HC), an unsupervised machine learning technique, to objectively identify clinopyroxene compositional clusters and their variations during this intense eruptive phase. Our results show that variations of monitoring parameters and eruption intensity are expressed in the mineral record both as changes in cluster proportions and the chemical complexity of single crystals. We also apply random forest thermobarometry to relate each cluster to P-T conditions of formation. We suggest that the February–April 2021 eruptive sequence was sustained by the injection of a hotter and deeper magma into a storage area at 1–3 kbar, where it mixed with a slightly more evolved magma. The February 28 episode emitted the most mafic magma, in association with the highest mean lava fountain height and highest time–averaged discharge rate, which make it the peak of the analyzed eruptive interval. Our results show that after this episode, the deep magma supply decreased and the erupted magma become gradually more chemically evolved, with a lower time–average discharge rate and fountain height. We propose this approach as a means to rapidly, objectively, and effectively link petrological and geophysical/geochemical monitoring during ongoing eruptions. We anticipate that the systematic application of this approach will serve to shed light on the magmatic processes controlling the evolution of ongoing eruptions.

**Supplementary Information:**

The online version contains supplementary material available at 10.1007/s00445-023-01643-2.

## Introduction

Trans-crustal magmatic processes and chemico-physical variations within volcanic plumbing systems profoundly influence the style and size of eruptions (Bachmann and Bergantz 2008; Cassidy et al. 2018). Understanding these parameters and their temporal evolution represents a key step in improving our understanding of volcanic activity and the ability to interpret pre-eruptive monitoring signals (Alparone et al. 2003; Pichavant et al. 2009; Andronico and Corsaro 2011). Mineral chemistry represents an essential archive recording pre and syn-eruptive processes, as minerals record their growth conditions by changing chemistry (Ginibre et al. 2007; Blundy and Cashman 2008; Costa 2008; Ruprecht et al. 2012; Cashman and Blundy 2013; Mollo et al. 2013; Barboni and Schoene 2014; Zellmer et al. 2016; Petrone et al. 2016; Cheng et al. 2017; Morgavi et al. 2017; Probst et al. 2018; Ubide et al. 2019; Caricchi et al. 2020; Weber et al. 2020; Sheldrake and Higgins 2021; Higgins et al. 2021; Boschetty et al. 2022). However, to quantitatively link monitoring parameters with processes occurring at depth, it is necessary to objectively identify chemically distinct zones in minerals. Such objective classification also allows for the intercomparison between eruptions, which is otherwise complicated by a degree of subjectivity of analyses based exclusively on human judgment. Without further delay, we state here that this approach is in no way a substitute for petrographic analysis and expert judgment. Machine learning is just a tool to enhance our interpretative capacity.

We focus on clinopyroxene because it is commonly found in mafic to intermediate magmas and crystallizes over a wide range of temperatures (T) and pressures (P; Hirschmann et al. 2008; Putirka 2008; Petrone et al. 2016; Ubide et al. 2019). Clinopyroxene chemistry is also sensitive to changes in magma composition, water content, pressure, and temperature (Putirka 2008; Mollo et al. 2018; Ubide et al. 2019). Secondly, the overall low diffusion rate of chemical constituents in its crystal lattice effectively preserves a record of the growth conditions (Müller et al. 2013; Petrone et al. 2016; Ubide and Kamber 2018; Ubide et al. 2019). Indeed, it has been demonstrated how Fe and Mg diffuse slower in clinopyroxene compared with other mafic minerals (Müller et al. 2013, Ubide and Kamber 2018). Additionally, of the elements in a clinopyroxene Fe and Mg are some of the fastest diffusing elements, meaning that other elements have even lower diffusivities (Müller et al. 2013; Ubide and Kamber 2018; Lierenfeld et al. 2019). These properties make clinopyroxene not only an excellent tracer of the chemical evolution of magmatic reservoirs (Winpenny and Maclennan 2011; Ubide and Kamber 2018; Caricchi et al. 2020; Boschetty et al. 2022), but also a valuable thermobarometer (Putirka et al. 1996, Nazzareni et al. 2001; Putirka 2008; Mollo et al. 2015; Petrelli et al. 2020; Higgins et al. 2022; Nazzareni et al. 2022; Jorgenson et al. 2022).

Mt. Etna volcano is intensively monitored by the Istituto Nazionale di Geofisica e Vulcanologia-Osservatorio Etneo (INGV-OE) via the collection of geochemical, volcanological, geophysical, and satellite monitoring data, together with a regular sampling of all eruptive events (Corsaro and Miraglia 2022). Thus, the paroxysmal sequence of the South-East summit crater of Etna from February to April 2021 provides an attractive opportunity to link petrology, monitoring signals, and eruptive parameters (such as, the repose time before the eruptive event, the mean fountain height, the erupted volume during the fountaining activity, the time-averaged discharge rate and the cumulative reduced displacement). To this aim, we analyzed the lava fountain episodes of February 16, 19, and 28 and March 2 and 10, when the eruptive frequency was relatively high (one episode every 1–3 days). Additionally, the presence of abundant literature data permits the comparison with past eruptions (Behncke and Neri 2003; Di Renzo et al. 2019). In general, continuous monitoring and existing data make Mt. Etna a uniquely suitable candidate for studying the temporal evolution of plumbing system processes and their influence on the eruptive behavior.

## Geological setting

**Fig. 1:**
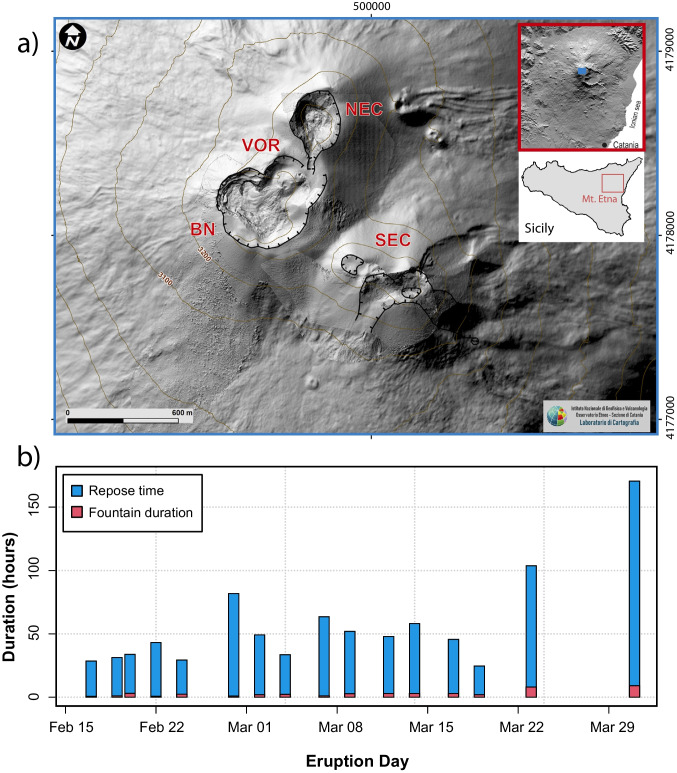
**a** Digital elevation model (DEM) of the Mt. Etna summit area, image modified from Corsaro and Miraglia, 2022. The red rectangle indicates the position of Mt. Etna in Sicily, the blue rectangle the location of the summit area on Etna; **b** bar plot of the paroxysmal events occurred at SEC between February 16 and April 1. Blue represents the repose time before the eruptive event and red represents the duration of the lava fountain activity (Calvari and Nunnari 2022)

Mt. Etna is located in eastern Sicily (Italy; Fig.1a). Its >3300 m elevation and 1250 km^2^ areal extent make it the largest active European volcano (Branca et al. 2004). It is also one of the most active volcanoes in the world (Cappello et al. 2013; Corsaro and Miraglia 2022). Mt. Etna volcano presents a wide range of eruptive behaviors, from purely effusive to more explosive eruptions, including strong strombolian and violent lava-fountaining events (Branca and Del Carlo 2004; Ferlito et al. 2014; Corsaro and Miraglia 2022). It produces eruptions from fissure vents along its flanks and its summit craters (Branca and Del Carlo 2004; Di Renzo et al. 2019). For both types of eruptions, a significant increase in volcanic activity has been observed over the last decades (Behncke and Neri 2003; Branca and Del Carlo 2005), in particular since 1971, with the formation of the South-East Crater (Cappello et al. 2013). The summit area of the volcano is composed of four active vents (Fig. 1a): Voragine (VOR), Bocca Nuova (BN), North-East Crater (NEC), and South-East Crater (SEC). Among these, SEC represents the youngest but also the most active crater (Andronico and Corsaro 2011; Di Renzo et al. 2019; Corsaro and Miraglia 2022). SEC has often produced cyclical or episodic eruptive activity (Parfitt and Wilson 1994; Spina et al. 2019), characterized by a series of paroxysmal lava fountaining events with very short repose times (Andronico and Corsaro 2011; Spina et al. 2019; Corsaro and Miraglia 2022). This is exemplified by 23 lava fountain episodes in 1998 (Neri et al. 2011), the 64 paroxysms in 2000 (Andronico and Corsaro 2011), and the 44 fountaining events between January 2011 and December 2013 (Calvari et al. 2018; Bonaccorso et al. 2021).

A new cyclical eruptive phase started at the SEC on December 13, 2020, producing over 60 paroxysms up to February 21, 2022 (Andronico et al. 2021; Bonaccorso et al. 2021; Marchese et al. 2021; Calvari and Nunnari 2022). The activity went through a relatively intense sequence from February 16 to April 1, 2021, with a paroxysm occurring every 1.85 days (± 0.67 days) until March 19 and longer repose times for the last two events (March 23–24 and March 31–April 1, Fig. 1b) for a total of 17 paroxysmal events (De Gori et al. 2021; Marchese et al. 2021; Calvari and Nunnari 2022; Corsaro and Miraglia 2022). Each paroxysmal event followed the typical pattern observed at the SEC in recent decades (Alparone et al. 2003; Andronico and Corsaro 2011; Calvari et al. 2011, 2021; Calvari and Nunnari 2022; Corsaro and Miraglia 2022), with an intensification of strombolian activity which has culminated in fountaining event (Calvari and Nunnari 2022; Corsaro and Miraglia 2022). During the fountaining activity, a sustained eruption column was generated, which could extend to several kilometers in height (Andronico et al. 2021; Calvari et al. 2021; Calvari and Nunnari 2022). As shown in Fig. 1, the sustained fountaining events between February and April 2021 lasted from about 51 min to a maximum of ~ 13 h, with a median duration of about 2.7 h (lava fountain duration data from Calvari and Nunnari 2022; Fig. 1b).

## Materials and methods

Small lapilli (maximum long-axis length 2 cm) were sampled from the tephra fallout deposits of the 16, 19, and 28 February and 2 and 10 March lava fountains. From each eruption, randomly selected lapilli were prepared for chemical analysis.

### Electron probe micro analyzer (EPMA)

Major element analyses of clinopyroxene crystals and glass were collected using a JEOL 8200 Superprobe at the University of Geneva and a JEOL JXA-8530F at the University of Lausanne. Clinopyroxene analyses were collected with a focused beam (diameter between 1 and 2 μm) at an accelerating voltage of 15 keV and a beam current of 20 nA. The glass analyses were conducted with either a 10-μm-diameter beam (15 keV, 6 nA) or a 5 μm diameter (15 keV and 4 nA) depending on glass pool size.

Calibration for clinopyroxene quantitative analyses was performed using the following standards: forsterite for Mg, fayalite for Fe, Mn-Ti oxide for Mn and Ti, albite for Na, wollastonite for Si and Ca, orthoclase for Al, NiO for Ni, and chromium oxide for Cr. Chemical analyses were collected along rim-to-core transects with a point spacing of ~ 2 μm for a total of 1250 analyses. Additional 29 analyses were collected for glass at various locations within the groundmass of each sample.

Analyses of clinopyroxene were filtered by removing all data with a total oxide content below 98% and above 101% and cations per formula unit higher than 4.04 and lower than 3.98 (recalculated assuming all iron in clinopyroxene to be FeO). Chemical analyses of clinopyroxenes and glass are available in the supplementary materials table (Supplementary Tables 1 and 3).

### Data transformation

A geochemical dataset (like the EPMA data used in this study) can be defined as a “closed” dataset. A dataset is considered “closed” when the variables it contains, in our case the oxide concentration, are not independent but are related in some way to each other. In this context, the oxides are expressed as wt%, so their nominal sum is 100% (Templ et al. 2008). Conducting statistical analysis directly on “closed datasets” can lead to issues (Butler 1976; Aitchison 1986; Templ et al. 2008) as some statistical approaches require the data to be normally distributed and not constrained to a constant total value (Boschetty et al. 2022). Various data transformations have been proposed to solve this issue such as the additive log-ratio transformation, the centered log-ratio transformation (Aitchison 1986), and the isometric log-ratio (ilr) transformation (Egozcue et al. 2003). Ilr has been shown to work well with geochemical data (Aitchison and Egozcue 2005; Templ et al. 2008; Filzmoser et al. 2009; Carranza 2011; Reimann et al. 2011; Boschetty et al. 2022). Ilr is a data transformation method that can be easily applied to a multivariate environment and has the particular property of representing the variables of our closed system in a real Euclidean space (Templ et al. 2008; Filzmoser et al. 2009; Carranza 2011). This method is useful to transform skewed data distributions into distributions closer to normal (Templ et al. 2008; Filzmoser et al. 2009). This property is useful for improving clustering results as shown by Boschetty et al. (2022). We apply the ilr transformation to our filtered data, using the formula proposed by (Egozcue et al. 2003), where the i components, transformed with the ilr function (*y*=ilr(*x*)), have been calculated as follows:

1 $${y}_i=\sqrt{\frac{i}{i+1}}\ln \left[\frac{g\left({x}_1,\dots, {x}_i\right)}{x_{i+1}}\right],i=1,2,\dots, D-1.$$

In Eq. 1, *D* are the parts of the compositional analysis (the number of analyzed elements for each spot analysis, *x*), *i* is one particular part of *D* (one of the ith analyzed elements, e.g., the wt% of SiO_2_) and g(*x*_i_) is the geometric mean of the ith parts of *x*, which in our case corresponds to the geometric mean of the analyzed elements, for example, from *x*_1_ = SiO_2_ to *X*_i_ = CaO (Egozcue et al. 2003).

Working with the ilr transformation requires non-zero values in the starting dataset (Cortés et al. 2007; Templ et al. 2008; Carranza 2011; Boschetty et al. 2022). The ilr transformation has been performed using the library “compositions” included in the open-source software R (R Core Team 2021). Zeros can occur in the investigated dataset for different reasons (Pawlowsky-Glahn and Olea 2004; Cortés et al. 2007; Boschetty et al. 2022). In the case of below-detection-limit zeros, replacement methods are available (Fry et al. 2000; Martín-Fernández et al. 2000; Boschetty et al. 2022), but from a general perspective, if zeros represent more than half of the studied dataset, the element in question should not be used for cluster analysis (Boschetty et al. 2022). In our case, most of the Cr_2_O_3_ analyses are below the detection limit; thus, this oxide was not considered for cluster analysis.

### Data normalization

An additional issue with geochemical data is the different ranges and absolute values over which the different oxides vary. Variability and absolute abundance may result in the least concentrated oxides overwhelmingly controlling cluster analysis (Templ et al. 2008; Caricchi et al. 2020; Boschetty et al. 2022). To circumvent this issue, the data were normalized with the median-mad method (Templ et al. 2008; Rousseeuw and Hubert 2011; Eesa and Arabo 2017). This method transforms the dataset by subtracting the median and dividing by the median absolute deviation (MAD; Huber 2004; Rousseeuw and Hubert 2011). The MAD is the median of the absolute deviations from the median and can be calculated as follows:

2 $$MAD= median\ \left\{\left|{x}_i-{M}_n\right|\right\},\kern0.5em with\ {M}_n= median\left\{{x}_i\right\}$$

Calculating MAD using Eq. (2) the final normalization formula will be:

3 $${X}^{\prime }=\frac{x_i-{M}_n}{MAD}$$

where *X*^′^ is the normalized dataset.

The median-mad normalization can be easily applied to the transformed dataset using the “stats” package in R (R Core Team 2021).

### Hierarchical clustering

Clustering methods are useful to subdivide multivariate observations into representative and homogeneous groups (Templ et al. 2008). Hierarchical clustering (HC) is an unsupervised learning method to identify clusters of similar chemical composition within and between crystals, by assigning each analysis to a cluster. This approach can recognize similar chemical sequences from the core to the rim of disparate crystals and help identify their chemico-physical growth conditions (Caricchi et al. 2020). This method has been already successfully applied to address geochemical and petrological problems (Templ et al. 2008; Caricchi et al. 2020; Boschetty et al. 2022) and can be implemented using the open-source software R, using the library “cluster” (R Core Team 2021). In detail, the standard implementation of the HC uses the Euclidean distance (d_ij_) of the filtered, transformed, and normalized dataset to estimate the similarity (or dissimilarity) among different analyses in the Euclidean space:

4 $${d}_{ij}=\sqrt{{\left({x}_i-{x}_j\right)}^2+{\left({y}_i-{y}_j\right)}^2 + \dots +{\left({z}_i-{z}_j\right)}^2},$$

where the variables from *x* to *z* represent the ilr components and *i* and *j* are the indexes of two different analyses. To cluster similar occurrences, different linkage criteria can be used. One of the most widely used criteria is the Ward minimum variance (Ward 1963). This method starts considering each analysis as an individual cluster. Then, it pairs similar occurrences into clusters, considering the smallest values of d_ij_ between them. It continues by progressively joining occurrences and incorporating within the clusters only the points which minimize average variance within clusters (Caricchi et al. 2020). Results can be visualized in a dendrogram. In the dendrogram, the branch length corresponds to the d_ij_ distance necessary to merge two distinct clusters into one.

### Outlier detection

In natural datasets, outliers (i.e., observations which deviate from the majority) are commonplace. In our case, this could result from the mixed analysis of two zones belonging to different compositional clusters (Rousseeuw and Hubert 2011). The identification of outliers is based on the difference (or distance) between a specific analysis and a cluster of analyses. The Mahalanobis distance provides such a measure (Mahalanobis 1936; Boschetty et al. 2022) and was used in this study to identify and remove outliers. The Mahalanobis distance for each observation is calculated as:

5 $${D}_M\left(\overrightarrow{x}\right)=\sqrt{{\left(\overrightarrow{x}-\overrightarrow{\mu}\right)}^T\ {C}^{-1}\ \left(\overrightarrow{x}-\overrightarrow{\mu}\right)}$$

where $\overrightarrow{x}$ represent a matrix of observation in a defined cluster, *C* is the center location estimator of the distribution, and $\overrightarrow{\mu}$ the covariance estimator. Following the procedure reported by Boschetty et al. (2022), we marked as outliers all observations that lie outside of the 97.5 percentile range of the distribution.

### Cluster visualization

**Fig. 2:**
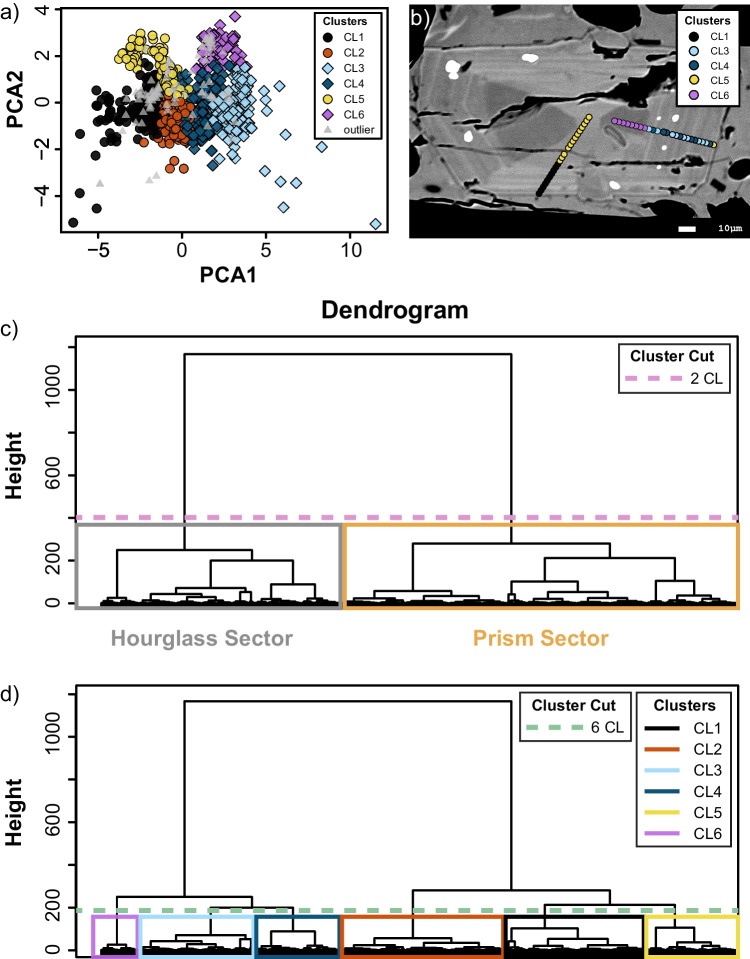
**a** Plot of the first and second principal components (PCA1 and PCA2) from the principal component analysis performed on the geochemical dataset and color contoured according to the number of clusters. **b** BSE image on a sector zoned cpx with the analyzed points colored according to the number of clusters. **c** and **d** dendrogram divided in 2 and 6 clusters, respectively

To visualize and validate the results of the cluster analysis, we apply principal component analysis (PCA), a dimensional reduction method (Hotelling 1933; Abdi and Williams 2010; Jolliffe and Cadima 2016; Boschetty et al. 2022), on the ilr-transformed dataset. PCA is a dimensional reduction method which allows for the visualization and interpretation of complex multivariate dataset while minimizing the loss of information (Hotelling 1933; Abdi and Williams 2010; Jolliffe and Cadima 2016; Boschetty et al. 2022). PCA allows us to visualize our multidimensional dataset (e.g., the chemical composition of the crystals, their oxide content, or in our case the six ILR-transformed vectors) in a smaller number of components representative of the multidimensional variance of our dataset (Hsieh 2009; Unglert et al. 2016; Bisciotti et al. 2022). In our case PCA1 and PCA2 explain alone up to the 92% of the total chemical variance (loadings, scores, and contributions of PCA analysis are reported in the Supplementary Table 4). If the clustering correctly identifies compositional clusters, they should be distinguishable when plotted in PCA1-PCA2 space, which is the case as shown in Fig. 2a. Additionally, we test the validity of the cluster analysis by visually inspecting if specific clusters correspond to textural features within crystals (Fig. 2b).

### Identifying the representative number of clusters

**Fig. 3:**
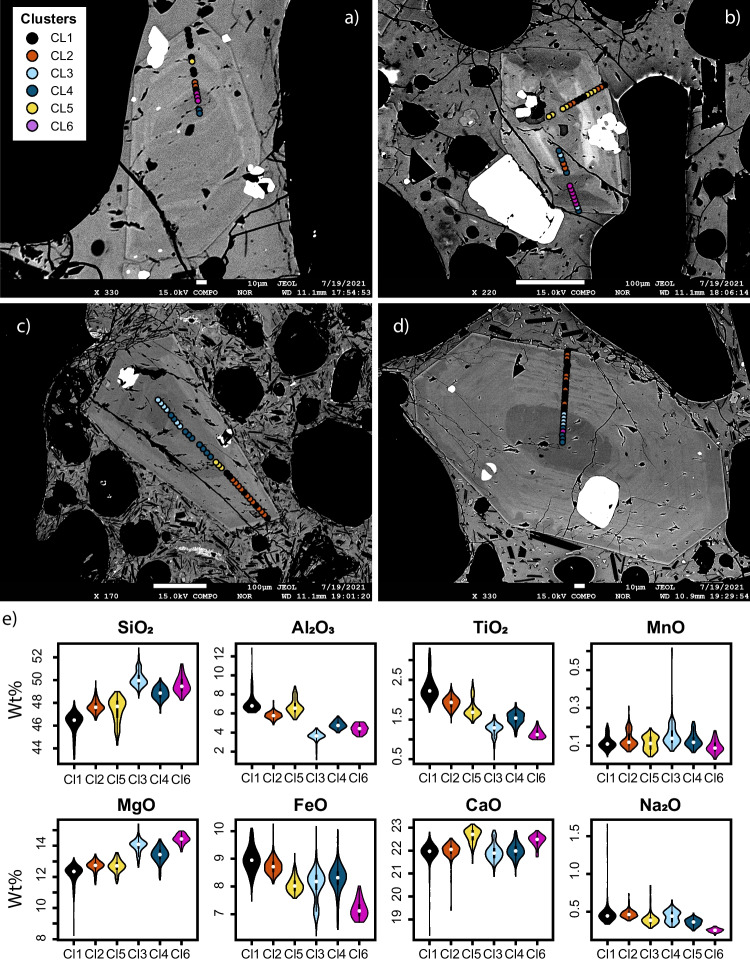
**a**, **b**, **c**, and **d** back scattered images of clinopyroxene from the February 16, 19, and 28 and March 02 in order. The analyzed points on the clinopyroxene are colored according to the number of clusters. **e** Violin plots of cluster composition for all the analyzed elements

Cluster analysis is an unsupervised learning method and there is no a priori correct number of clusters. Therefore, different strategies must be adopted to identify the relevant number of clusters for the Etna dataset. Several quantitative clustering validation techniques are available (Halkidi et al. 2001; Templ et al. 2008; Charrad et al. 2014; Sheldrake and Higgins 2021), although when dealing with geochemical datasets, they are insufficient to robustly determine the exact number of clusters (Templ et al. 2008, Supplementary document 1). The result of multivariate cluster analysis is a distance matrix in Euclidean space (Templ et al. 2008; Caricchi et al. 2020; Sheldrake and Higgins 2021; Boschetty et al. 2022). A good method to visualize the distance matrix is represented by a dendrogram, where the *y*-axis represents the distances between observations (Temple et al., 2008; Caricchi et al., 2020; Sheldrake and Higgins, 2021; Boschetty et al., 2022). A qualitative examination of the dendrogram can inform about the number of clusters within a dataset, whereby the dendrogram is cut at different heights to separate the dataset into different numbers of clusters (Fig. 2c, d). Templ et al. (2008) demonstrate that the number of clusters in a geochemical dataset should be assessed with respect to the known properties of the object of investigation. In this regard, we generated a series of results in an iterative fashion, increasing the number of clusters from two to eight. For each configuration, we first tested the chemical validity of the clustering by visualizing the data in PCA1-PCA2 space through color coding the points according to the relative clusters, allowing us to verify that each cluster was chemically distinct (Fig. 2a, Hotelling 1933; Jolliffe 2002; Abdi and Williams 2010; Jolliffe and Cadima 2016). Secondly, we ensured that each cluster corresponded to a texturally defined area of the crystal. A visual textural analysis was conducted by overlaying the analyzed points on the BSE images, assigning each point the color of the relevant cluster (Fig. 2b, Fig. 3a), and checking that each cluster corresponded to a texturally distinguishable area that was large enough not to be the result of mixing between two chemical zones. The match between the zoning pattern and clusters for selected crystals was an additional confirmation that the clustering identified geochemically meaningful groups of analyses (Fig. 3a–d).

### Random forest thermobarometry

We applied random forest (RF) thermobarometry (Petrelli et al. 2020; Higgins et al. 2022; Jorgenson et al. 2022) to the clinopyroxene chemical dataset to estimate the pressure (P) and temperature (T) at which crystals grew. RF is a machine learning approach capable of building a robust prediction that extends the capability of the decision trees algorithm (Breiman 2001; Simm et al. 2014; Jorgenson et al. 2022). In the decision trees algorithm (Breiman 2001), each tree is a hierarchical flowchart composed of numerous branches. Their structure is determined using training data, in our case the chemical composition of experimentally derived clinopyroxene (Hirschmann et al. 2008; Jorgenson et al. 2022). Each branch ends with a prediction of the investigated parameter (i.e., P or T). The decision tree algorithm can be applied to natural data by following the branch structure to arrive at P or T estimate. However, predictions based on single decision tree models are inaccurate due to so-called overfitting (Zhou et al. 2020). If overfitting occurs, the model can only accurately predict the experimentally derived clinopyroxene (training dataset) but not the natural samples (unknowns). Thus, a more robust model, like the RF, is required (Petrelli et al. 2020; Zhou et al. 2020; Jorgenson et al. 2022). Following the suggestions reported by Jorgenson et al. (2022), we develop a RF model characterized by 200 trees, resulting in 200 different predictions of the investigated parameter (i.e., P and T) for each unknown sample. For natural data prediction, we used the median of the voting distribution as the best P–T estimate. In our specific case, the RF algorithm was applied using the open-source R script available on GitHub (https://github.com/corinjorgenson/RandomForest-cpx-thermobarometer) provided by Jorgenson et al. (2022). The results of the hierarchical clustering and random forest thermobarometry analyses can be found in the supplementary material table (Supplementary Table 2)

### Random forest classifier

We finally test a RF classifier assigning clinopyroxene analyses from volcanic products that erupted from the SEC in the last two decades to the clusters we identified for the period between February and April 2021. We trained the algorithm using the clinopyroxene dataset of this work and applied it to clinopyroxene analyses for the eruptions of 2002–2003; 2006; 2007; 2008; 2011–2012, available on GEOROC (DIGIS Team 2022). This approach is useful to compare eruptions and eventually identify patterns between the fraction of different clusters and eruptive dynamics. The RF algorithm was trained on 200 trees. The performance of the method was tested by randomly removing 10% of the observations from the starting train dataset, for which the chemical clusters were known, and applying the model to this 10% by treating it as an unknown. The classifier correctly classified over 95% of the observations in the test dataset.

### Textural and chemical complexity of minerals

To quantify compositional and textural variations among and within different paroxysms, we define a new parameter, the textural complexity (tx_comp_), calculated as the number of times a change of cluster occurs from the rim to the core of a crystal (C_cl_) divided by the length of the analyzed profile (L_a_):

6 $${tx}_{comp}={~}^{{C}_{cl}}\!\left/ \!{~}_{{L}_a}\right..$$

We couple this parameter with the variance from rim to core for each oxide normalized for the length of the analyzed transect. These two parameters provide a quantitative measure of the textural and chemical complexity of each crystal.

To ensure the representativeness of these parameters, special attention must be paid to the crystal selection. The sectioning effect, i.e. how the 3D crystal was cut to produce the analyzed 2D section, is not negligible (Shea et al. 2015; Cheng et al. 2017). The random cuts of a crystal can be responsible for the presence of a wide variety of artifactual zonation (e.g., different cuts of crystals with the same number of zones — Cheng et al. 2017). To limit this effect, only the largest crystals (major axis > 80 μm) showing similar section cuts were used in the calculation of chemical and textural parameters. In this process, the presence of sector zoning helped to identify similar sections.

## Results

The analyzed lapilli have trachy-basaltic composition, and their mineral assembly is constituted of plagioclase+clinopyroxene+olivine+oxide phenocrysts (microcrysts having a length < 100 μm and mesocrysts with a length between 100 and 500 μm, Zellmer 2021; Mollo et al. 2022). The groundmass has differing proportions of microlites for each eruption, and the microlites are mostly composed of plagioclase and clinopyroxene. All the analyzed clinopyroxene phenocrysts show concentric oscillatory zonation, and almost all also show a clear hourglass zonation. The glass composition shows an increase in Al_2_O_3_ and MgO from February 16 until February 28, which then decrease in the 2 and 10 of March eruptive products (Figs. S1 and S4; Supplementary Table 3), in agreement with data from Corsaro and Miraglia (2022).

### Cluster identification

The dendrogram shows two clear groups of clusters (Fig. 2c, d). As shown in Fig. 2c, the two clusters texturally distinguish between hourglass sectors, and prism sectors as their chemical composition are the most compositionally contrasted. However, two clusters are insufficient to separate discrete cores and concentric zones. Six clusters provide the best partitioning of data from both chemical and textural perspectives as shown by the plot of the two principal components of PCA and the correspondence between the cluster and the zones identified in the BSE images (Figs. 2a, b and Figs. 3a–d). Attempting to partition the data in a larger number of clusters results in a lack of correspondence between cluster and textural features and in a lower quality of the chemical split in the PCA1–PCA2 space. This approach reinforces the importance of combining textural and chemical observations when describing zoned magmatic crystals (Sheldrake and Higgins 2021).

### Cluster chemistry

Three of the six clusters (CL1, 2, and 5) are found in some cores, in concentric zones within the prism sectors, and within outer growth rims. The other three clusters (CL3, 4, and 6) are only found in hourglass sectors, either in cores or mantles (Fig. 3a). Many of the analyzed crystals are cut perpendicular to the *c*-axis, where the hourglass sector occupies the center of the crystals (Leung 1974; Ubide et al. 2019). However, other crystals are cut so that only small portions of the hourglass zoning are visible. This results in an uneven number of points analyzed in the hourglass zone, which can vary from sample to sample. This non-uniform sampling could lead to misleading variations in the fraction of the hourglass clusters (CL3, CL4, and CL6) between different paroxysms. Thus, we only consider the analyses collected within the prism sector.

**Fig. 4:**
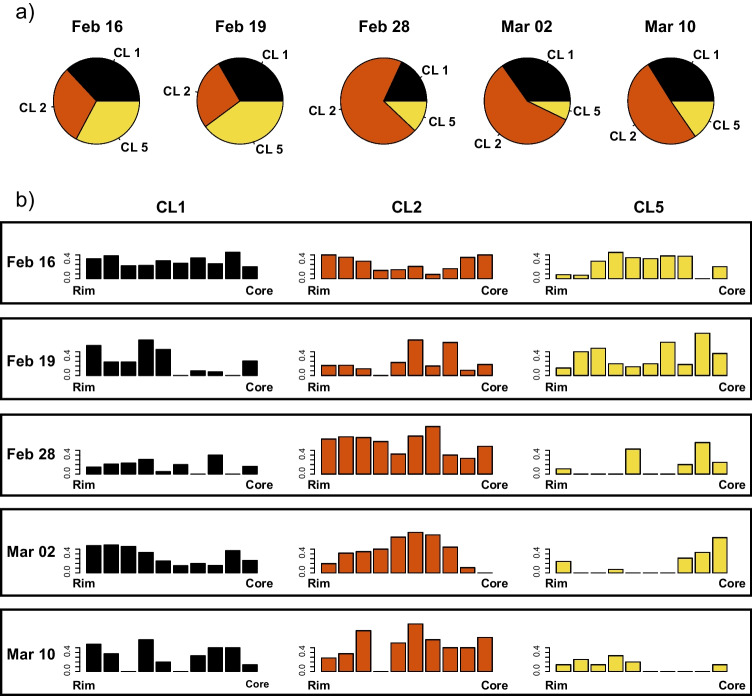
**a** Prism sector cluster distribution in different eruptive events. **b** cluster distribution between cores and rims of clinopyroxene, within all the studied episodes, normalized between 0 and 1

In general, the hourglass sectors present lower concentrations of Al_2_O_3_ and TiO_2_ (4.17 and 1.34 wt.% with respect to 6.42 and 2.01 wt.% in the prism sectors, for Al_2_O_3_ and TiO_2,_ respectively) and an enrichment in SiO_2_ and MgO (with a mean composition of SiO_2_ and MgO of 49.56 and 13.84 wt.% for the hourglass and 47.13 and 12.53 wt.% for the prism sectors) compared to the prism sector. The concentric zonation within prism sectors displays an alternation of CL1, CL2, and CL5 (Figs. 2b and Figs. 3a–d). In detail, comparing these three clusters with each other, it can be seen that CL1, among the three, exhibits a higher median content in FeO (8.95 wt.%), TiO_2_ (2.21 wt.%), and Al_2_O_3_ (6.78 wt.%), and a lower content in SiO_2_ (46.47 wt.%), CaO (21.97 wt.%), and MgO (12.35 wt.%). CL2 and CL5 present a higher and similar median content of SiO_2_ (47.6 wt.% for both) and MgO (12.74 wt.% for CL2 and 12.70 wt.% for CL5). While they are similar in SiO_2_ and MgO, CL5 show a higher content in CaO (CL5: 22.71 wt.%, CL2: 22.05 wt.%) and Al_2_O_3_ (CL5: 6.57 wt.%, CL2: 5.77 wt.%) and lower FeO (CL5: 8.02 wt.%, CL2: 8.71 wt.%) and TiO_2_ (CL5: 1.68 wt.%, CL2: 1.93 wt.%; Fig. 3e). The pie charts in Fig. 4a shows the distribution of CL1, CL2, and CL5 through different lava fountains. Analyses collected on clinopyroxene erupted on February 16 and 19 show a similar proportion of the three clusters consisting of an almost equal amount of CL1 and CL5, and minor content of CL2. The CL2 content increases abruptly in clinopyroxene from the February 28 event, before decreasing gradually through the March 2 and 10 paroxysms (Fig. 4a).

### Cluster distribution within crystals

The variations of the relative proportions of clusters over time are associated with changes in the distribution of clusters from the core to the rim of the crystals (Fig. 4b). For the February 16 episode, the majority of the rims are CL2 or CL1 in an almost equal amount, the mantle is mainly constituted of CL5 and the core of CL2 or CL1. In the February 19 crystals, the rims are mainly CL1, the middle part is mostly an alternation of CL1 and CL2, and the cores are predominately CL5.

The February 28 paroxysm shows more uniform crystals, constituted mostly of CL2; CL5 can be found mainly in the cores and in the middle portion of the crystals, and CL1 is in all crystals as a small concentric zone. Most of the March 2 clinopyroxene presents CL1 in the rims and CL2 in the central portion (mantles), with CL5 as a dominant phase in cores. Lastly, the March 10 clinopyroxene presents an oscillating distribution of CL1, which alternates with CL5 and CL2 in complex concentric zoning from rim to core. CL2 can be found everywhere from the rim to the core and CL5 mainly in the rims. Overall, we can observe a shift from rim to cores of CL5 from February 16 to the event of March 2, and CL5 mostly in the rim in the March 10 event. CL1 usually represents the rims, with the exception of the February 16 paroxysm where its distribution is generally constant through the profiles, and the March 10 event where an oscillating distribution is observed. CL2 can be found both in cores and in alternation with CL1 and CL5 in rims and in the central part of the crystals, and it is the dominant phase everywhere on February 28.

### Random forest thermobarometry

**Fig. 5:**
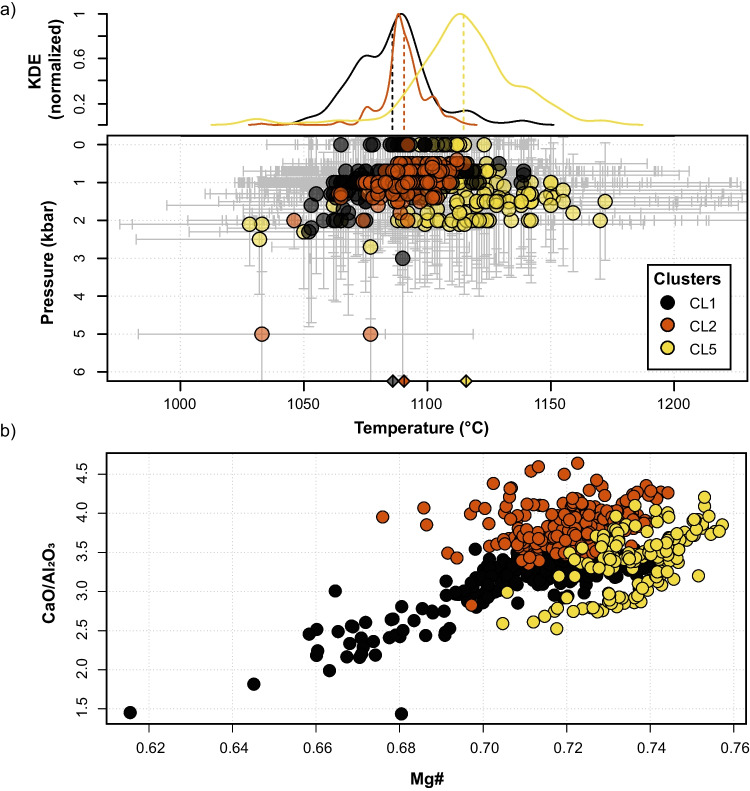
**a** P and T estimates for each analyzed point, color contoured according to the cluster number, the error bars are based on the interquartile range. On top of the plot, the kernel density estimate normalized between 0 and 1 show the density distribution of T estimates for the three clusters. **b** Magnesium number (Mg#) plotted vs CaO/Al_2_O_3_

The random forest thermobarometry results for our dataset are shown in Fig. 5a, where the estimates of P and T median values are plotted and color-contoured according to their respective clusters. Most analyses record a pressure between 1 and 3 kbar (SEE = 2.6 kbar) and a temperature between 1070 and 1130 °C (SEE = 58.9 °C). Such values agree with literature estimates of Etnean magma storage (Murru et al. 1999; Spilliaert et al. 2006; Giacomoni et al. 2014; Mollo et al. 2022). While the pressure is similar for the three clusters, they seem separated in terms of temperature, with CL1 recording the lowest temperatures and CL5 the highest. Even if the single estimates are within the uncertainty of our approach, differences are still visible in the distributions (Fig. 5a). While CL1 presents lower temperatures and more evolved compositions, CL2 and CL5 register higher temperatures and show a similar Mg# (Fig. 5a, b). CL2 shows higher CaO/Al_2_O_3_ values with respect to CL5. The Mg# and the crystallization temperature reach higher values in CL5 clinopyroxenes. (Fig. 5).

### Random forest cluster classifier

**Fig. 6:**
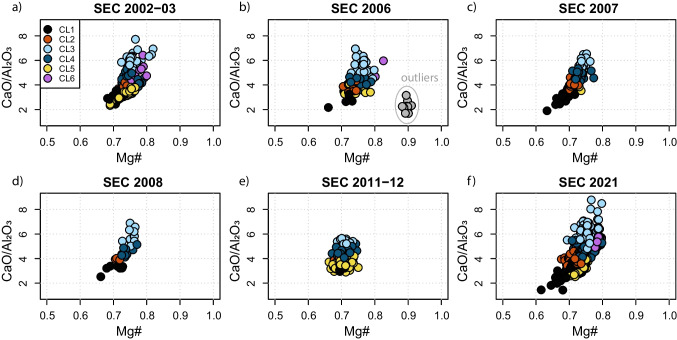
Mg# vs CaO/Al_2_O_3_ composition of clinopyroxene from different SEC eruptions. The colors of the points represent the cluster estimated by an RF classifier which was trained on the dataset from this paper. Circled in light gray are the chemical outliers revealed in the chemical analysis of cpx from the 2006 eruption

We tested the potential of a random forest classifier to identify clusters in previous eruptions, using the data we collected in 2021 as a training dataset. The results of the random forest cluster classification on previous SEC eruptions are shown in Fig. 6. The clusters identified for the 2021 eruptive sequence describe sufficiently well the chemical variability of past eruptions, with the exception of a group of data in the 2006 eruption. The chemistry of this group of clinopyroxenes is in agreement with xenocrystals, potentially in equilibrium with the mantle (Fig. 6b). Even if the xenocrystic nature of these crystals would require petrographic confirmation, this represents a good example of something that might happen during an eruption when clinopyroxenes of a previously unrecognized group are analyzed. To construct a reliable geochemical classifier for all Etna crystals, it is hence necessary to build a larger geochemical dataset that collects all existing clinopyroxene analyses and a description of their textural location. Once such a dataset will be constructed, the classifier we present here will allow the identification of the cluster for each collected analysis in clinopyroxene and therefore the rapid quantification of the proportions of different clusters in the erupted material. This approach would have three major advantages: (1) the rapid quantification of the proportions of analyses belonging to different clusters; (2) the quantitative comparison with previous eruptive events; (3) anticipate the evolution of an ongoing eruptive sequence on the base of previously identified quantitative temporal trends in the fraction of analyzed clusters.

## Discussion

### Clusters as recorders of the evolution of crystallization conditions

The February 28 events have the lowest counts for the lower temperature cluster (CL1) and thus the highest amount of analyses assigned to CL2 and CL5 (Fig. 4a). The increased contribution of mafic magma to this event is confirmed by the glass analyses (Fig. S2 and Supplementary Table 3), showing a peak of CaO/Al_2_O_3_ and MgO content on February 28. This trend was also observed by Corsaro and Miraglia (2022), who analyzed the glass composition of the SEC 2021 eruptive products over a wider time span (Dec 2020–Apr 2021) showing the same peak of higher mafic compositions on February 28. The observed trends in glass and crystal-chemical variation suggest the interaction and mixing between an increasing proportion of a hotter and more mafic melt with a slightly colder and more evolved magma until February 28, followed by a decrease of the hot magma input. This hypothesis is also confirmed by the observed clinopyroxene concentric zonation, which could be an expression of magma mixing (Ubide and Kamber 2018; Ubide et al. 2019; Mollo et al. 2022).

**Fig. 7:**
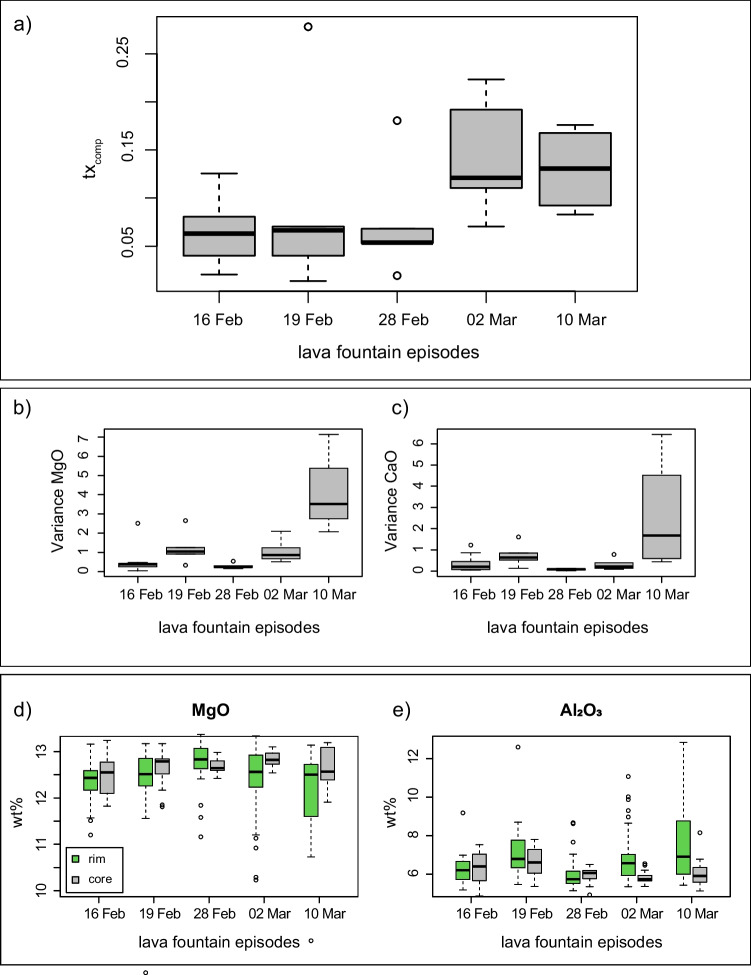
Box plots of: **a** textural complexity; **b** and **c** MgO and CaO variance for each episode; **d** and **e** MgO and Al_2_O_3_ clinopyroxene content in rim and cores for each paroxysm

We suggest that declining rates of mafic magma input after February 28 are reflected by increasing fraction of lower temperature CL1 clinopyroxene analyses. This is also confirmed by the core-to-rim distribution of clusters and the increasingly evolved nature of clinopyroxene rims after the episode of February 28 (Fig. 7b).

Mollo et al. (2022) studied the growing conditions of clinopyroxene during the same eruptive period, giving us a broader view of the fountaining sequence. These authors note that clinopyroxene that erupted on February 28 records higher crystallization temperature compared with the previous events, in agreement with our observations. They also analyzed crystals from the final two episodes of the eruptive sequence (March 24 and 31), where clinopyroxene crystallization temperatures were similar to those that erupted on February 28. Altogether these results suggest that the period from February to the end of March was characterized by two major events of input of mafic magma from depth at the end of February and at the end of March 2021.

While the chemical characteristics and estimated temperature for CL1 can be reconciled with the crystallization of these clinopyroxenes at relatively low temperatures in a cooling superficial reservoir, it remains unclear which process can produce the difference in CaO/Al_2_O_3_ and Mg# observed between CL2 and CL5. While more data would be required, we provide in the following a speculative interpretation. Looking at the thermobarometry result obtained from clinopyroxene chemistry, the lack of high-pressure estimates is evident (Fig. 5a). This can be either the product of lack of crystallization or resorption. Considering the water content of magmas at Mt. Etna (Métrich et al. 2004), their liquidus temperature increases with increasing pressure from a few kilometer depth. As the liquidus phase for these magmas is clinopyroxene (Fig. S3; calculations performed with rhyolite-MELTS; Gualda et al. 2012), magmas extracted deeper will have lower CaO/Al_2_O_3_ content because of more extensive clinopyroxene fractionation with respect to magmas extracted shallower at the same temperature. Thus, we could speculate that CL2 clinopyroxene crystallized from a melt extracted from a shallower depth with respect to the melt from which CL5 clinopyroxenes crystallized (Fig. 5b).

A second possibility is that partial resorption of clinopyroxene upon ascent resulted in an increased CaO/Al_2_O_3_ content of the melt from which CL2 clinopyroxenes crystallized at shallower depths. In this scenario, clinopyroxenes from CL5 crystallized from a melt that was extracted near liquidus, and therefore, no resorption could occur. This would agree with the higher temperatures recorded by clinopyroxenes from CL5 (Fig. 5a). The resorption of about 1.2 wt.% of clinopyroxene is sufficient to explain the difference in the CaO/Al_2_O_3_ in the glass between the 28 of February and the other fountaining events (Fig. S4).

### Cluster variabilities and eruptive dynamics for the February 28 episode

The proportion of clusters in samples from the 28 of February lava fountain is clearly different from the other episodes we investigated (Fig. 4a). Additionally, this event shows the lowest and least variable textural complexity of all eruptions, together with the lowest oxide (e.g. MgO and CaO) variance (Fig. 7a, b, and c). Hence, our results suggest that the February 28 event was fed by a more mafic magma, which potentially ascended faster (quasi adiabatically leading to an increase of the fraction of high CaO/Al_2_O_3_ CL2 clinopyroxenes). The lower textural and chemical complexity (low oxide variance along the crystal profile) of clinopyroxene from this event could be related to the growth of crystals in a more homogeneous environment with a larger volume of mafic magma. The presence of greater amounts of mafic magma is also confirmed by the results of our glass analyses (Fig. S2). The eruption of February 28 also shows a greater proportion of microlites, an observation confirmed by the CSD analyses conducted by Mollo et al. (2022). This could be due to a higher nucleation rate caused by the higher ascent velocity of a more mafic and hotter magma (Vona and Romano 2013; Vetere et al. 2022).

A comparison between clinopyroxene core and rim chemistry through the entire eruptive sequence shows that cores are more chemically homogeneous with respect to rims and the chemical variance of rims reaches a minimum in the products of February 28 before increasing again until the event of March 10 (Fig. 7d, e). This suggests that progressive input of mafic magma led to the most mafic lava fountain of February 28, after which the input of mafic magma declined, allowing for the growth of rims reaching the most chemically evolved composition with the last event we investigated. Existing data (Mollo et al. 2022) suggest another increase of the mafic input from depth occurred at the end of March 2021.

### Linking quantitative petrology to eruptive dynamics

Differences in the rate of magma input, chemistry, and temperature of the erupted magma can affect the eruptive dynamics because of the control of these intensive parameters on magma viscosity (Giordano et al. 2008). To investigate this hypothesis, volcanological and geophysical parameters were collected from the INGV multidisciplinary weekly bulletins (INGV-OE, Bollettini Settimanali sul monitoraggio vulcanico, geochimico e sismico del vulcano Etna at: https://www.ct.ingv.it/index.php/monitoraggio-esorveglianza/prodotti-del-monitoraggio/bollettini-settimanalimultidisciplinari) and from published literature (Andronico et al. 2021; Calvari and Nunnari 2022). Specifically, we analyzed the variation of repose time before the eruptive event, the mean fountain height, the erupted volume during the fountaining activity, the time-averaged discharge rate (TADR), and the cumulative reduced displacement (RD_cum_) between the 5 eruptive episodes studied in this work. RD_cum_ is the root mean square (RMS) of the volcanic tremor amplitude normalized for the distance of the measuring station from the source (Andronico et al. 2021). The RD value is cumulative and so accounts for values of volcanic tremor amplitude collected over a well-defined time window in which the tremor was recorded (Andronico et al. 2021). TADR represents the flux of volume of erupted tephra averaged over a specific time interval (Harris et al. 2007; Calvari and Nunnari 2022).

**Fig. 8:**
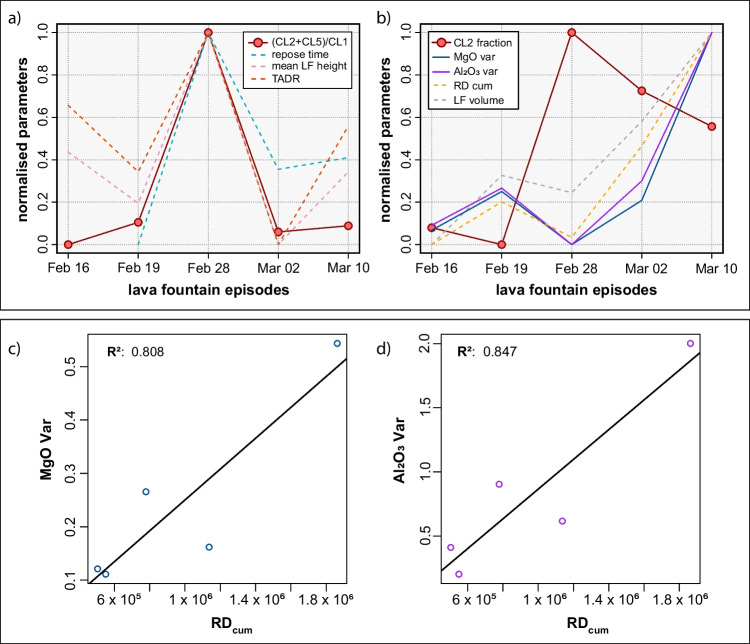
**a** Ratio between the mafic (CL2 and CL5) and more evolved (CL1) cluster fractions plotted for each lava fountain episode and compared with volcanological parameters such as: the repose time before the eruptive event, the mean lava fountain (LF) height, and the time-averaged discharge rate (TADR). All the parameters have been normalized, the information about repose time has been collected thanks to the multidisciplinary weekly bulletin available on the INGV website, while mean LF height and TADR have been taken from Calvari and Nunnari (2022). **b** The fraction of cluster 2 and the normalized variance of MgO and Al_2_O_3_ in clinopyroxene are plotted for each paroxysm and compared with the RD_cum_ (the RD_cum_ data has been taken from Andronico et al. 2021) and the LF erupted volume (from Calvari and Nunnari 2022). All the parameters have been normalized. **c** and **d** Regression lines and *R*^2^ are reported to mark the relationship between RD_cum_ and the MgO and Al_2_O_3_ variance from core to rim of the crystals

This analysis shows that the fraction of mafic magmas (i.e., clinopyroxenes from CL2 and CL5), which peaks on the 28 of February, follows a similar pattern to repose time, mean height of the lava fountain, and TADR (Fig. 8a). A good correlation is observed between the variance of MgO and Al_2_O_3_ in clinopyroxene, RD_cum_ and the volume erupted by the lava fountain (Fig. 8b). Finally, we observe an anticorrelation between the fraction of CL2 and RD_cum_ (Fig. 8b). These quantitative relationships should be further explored and potentially implemented for effective petrological monitoring in periods of protracted magmatic activity.

In agreement with the results of La Spina et al. (2021), our results show that the hotter and more mafic magma (i.e., lower viscosity) erupted on 28 February 2021, which led to the highest lava fountain event and TADR of the investigated period. The lower magma viscosity of this event probably caused faster rates of magma ascent. Moreover, higher rates of ascent (corresponding to a higher magma flow) increase crystal nucleation (Vona and Romano 2013), which would account for the more abundant microlite content of the magmas that erupted on February 28 (Fig. 3c and supplementary Fig. S1).

### A model for the February–April SEC eruptive sequence

We suggest that the paroxysmal activity which occurred during the February–April 2021 eruptive sequence at Etna was driven by the injection of mafic and deep magma into a region of the plumbing system that extends over a pressure range of 1–3 kbar, as suggested by our barometric estimates, some of which erupted to the surface. Here, new magma is mixed with slightly more evolved magma left from previous eruptions in January (Corsaro and Miraglia 2022). The mixing explains the complex concentric zoning in the rim characterized by alternating CL1 and CL2. After the February 28 eruption, the magma supply to the storage area decreased, and the occurrence of fractional crystallization processes produced magmas that gradually evolved to compositions comparable to those produced in January 2021. The February 28 eruption represents the peak of the February-April eruptive series involving the highest portion of mafic and hotter magma, and it is characterized by a higher ascent rate and fountain height.

### The RF cluster classifier

We have shown and validated on a known dataset that using a clustered dataset is a useful tool for robustly identifying discrete chemical clusters, which in turn can be related to magmatic processes as well as volcanological and geophysical monitoring parameters. We suggest that the RF cluster classifier method has the potential to recover the eruptive dynamics of past eruptions and to quickly establish a link between petrology and volcanological parameters.

To demonstrate the wider applicability of our method to historical eruptions, and to compare the chemical variability of clinopyroxene crystals measured in this work with those of past eruptions, we investigate a large dataset of clinopyroxene chemical composition from Mt. Etna, taken from GEOROC (DIGIS Team 2021). In Fig. 6, we see that the RF classifier distinguishes the proportions of clusters present in products from different eruptive periods. This result underlines the capability of this method to be used as a tool for reconstructing the chemical variations over time as recorded by crystals. To link mineral chemistry and eruptive dynamics, as we did in this study, the exact date of sample collection is necessary. However, this is not always available in the GEOROC database, where often only the year of the eruption is reported. As an example, in 2000, there were 64 lava fountain episodes (Andronico and Corsaro 2011). The connection between mineral chemistry and eruptive dynamics using our approach is thus only possible for samples for which the exact collection date is reported. Nevertheless, the results obtained on historical eruptions allow us to make several conclusions: first, we note that while six clusters might be enough to describe the chemical variability of products erupted by Mt. Etna over the last 20 years, an additional cluster for xenocrystals is necessary (Fig. 6). Additionally, our method can easily discriminate geochemical clusters and identify whether the analysis was collected on the prism or on the hourglass section. Finally, we propose a set of guidelines to make our approach more robust for quantitative petrological monitoring. During prolonged eruptions, the comparison should be facilitated by the collection of samples with similar characteristics (e.g., tephra or lavas for which the emission date is known) and a consistent analytical approach to keep track of the zone of the crystals in which the chemical transects and/or spot analysis are acquired (i.e., core and rim). Clearly, the assembly of a larger dataset collected with the sampling and analytical strategy we describe above will serve to improve the accuracy of our approach in linking the chemical evolution of minerals and eruptive dynamics.

## Conclusions

This study shows that the application of robust data transformation and normalization combined with HC analysis on geochemical datasets is a useful tool for identifying clusters of similar chemical-textural zones in minerals. This, in tandem with RF thermobarometry, can be used to trace specific P-T-X growth conditions and constrain the distribution of clusters on inter-eruption and intra-eruption base. The HC analysis on clinopyroxene underlines the presence of six chemically and texturally distinct clusters within the February 16 to April 1 Etna eruptive sequence. CL3, CL4, and CL6 are related to hourglass zones and CL1, CL2, and CL5 are related to prism sectors and to the concentric zoning. CL2 and CL5 represent a more mafic endmember, which proportionally increases up to the February 28 eruptive event before then decreasing. The application of RF thermobarometry to the clustered dataset allowed us to relate chemical clusters to P and T conditions. We estimate clinopyroxene crystallization to occur in a pressure range of 1–3 kbar and in a temperature range of 1070 and 1130 °C, in line with previous studies. CL1, CL2, and CL5 formed in the same pressure range but at different temperatures, where CL1 is a low-T cluster, and CL2 and CL5 are high-T clusters. We conclude that the 2021 Etna eruptive sequence was sustained by the intrusion of deep mafic magma into a storage area located at 1–3 kbar. Here, the mixing between a new fresh magma and a more evolved magma remaining from previous eruptions in January took place.

RF algorithms offer an effective and rapid way to classify and study the distribution of unique chemical clusters in different eruptive events. This represents a useful step in identifying the emergence of chemical compositions related to specific eruptive dynamics during an ongoing eruptive series, bringing a further contribution to the importance of syn-eruptive petrological monitoring. This has the potential to unveil magmatic processes at a depth that can control eruptive dynamics. More generally, the methodology shown in this work represents a fundamental tool for analyzing chemical variations in a robust and quantitative manner. This approach offers the possibility of quantifying chemical variations both within and between different eruptive events, allowing the direct correlation of the chemical variations with other monitoring parameters and enabling the construction of new parameters capable of tracing the chemical and textural complexity recorded by crystals. Moreover, we have shown the use of this technique on 1D profiles with its limitations. Extending the approach to 2D maps, in the future, could lead to a quantitative identification of zoning patterns and crystal families that underwent similar growth histories (e.g., Jerram and Martin 2008; Cheng et al. 2017; Sheldrake and Higgins 2021; Higgins et al. 2021). Finally, the use of clustering in conjunction with thermo-barometric methods allows rapid correlation between representative chemical compositions and their forming conditions.

## Supplementary Information

ESM 1 (PDF 589 KB)

ESM 2 (XLSX 196 KB)

ESM 3 (XLSX 451 KB)

ESM 4 (XLSX 15.3 KB)

ESM 5 (XLSX 134 KB)

ESM 6 (PDF 338 KB)
