# Supplementary material for: The magmatic evolution of South-East Crater (Mt. Etna) during the February–April 2021 sequence of lava fountains from a mineral chemistry perspective
Source: Bull Volcanol. 2023 Apr 26;85(5):33. doi: 10.1007/s00445-023-01643-2 (PMC10133385; doi:10.1007/s00445-023-01643-2)
Supplement: Supplementary file 1 — ESM 1 (PDF 589 KB) [file 445_2023_1643_MOESM1_ESM.pdf]

Supplementary Document 1

Bulletin of Volcanology (**Online Resource 5**)

Alessandro Musu1*, Rosa Anna Corsaro2, Oliver Higgins4, Corin Jorgenson1, Maurizio Petrelli3, Luca Caricchi1

1) Department of Earth Sciences, University of Geneva, rue des Maraîchers 13, 1205, Geneva, Switzerland
2) Istituto Nazionale di Geofisica e Vulcanologia, Osservatorio Etneo-Sezione di Catania, Catania, Italy.
3) Department of Physics and Geology, University of Perugia, Piazza dell'Università, 1, 06123 Perugia, Italy.
4) Geology, School of Natural Sciences, Trinity College Dublin, Dublin, Ireland
*Corresponding author (alessandro.musu@unige.ch; ORCID iD: 0000-0001-5354-5782)

How to identify the most representative number of clusters

Clustering analysis is an unsupervised learning method and there is no "a priori" number of clusters that is correct. Therefore, different strategies must be adopted to identify the number of clusters that is the most appropriate for the dataset under investigation. The result of multivariate clustering analysis is a distance matrix in the Euclidean space (Temple et al. 2008; Caricchi et al. 2020; Sheldrake and Higgins 2021; Boschetty et al. 2022). To estimate the ideal number of clusters, a qualitative analysis of the distance matrix can be performed by merging in the same cluster observations having a similar average distance (Sheldrake and Higgins 2021). A good method to visualize the distance matrix is represented by a dendrogram, where the y-axis represents the distances between observations. A qualitative examination of the dendrogram is a good approach to get a general idea of the number of clusters contained in a dataset. In detail, cutting the dendrogram at different heights allows to separate the dataset in different number of clusters (Fig. D1c and D1d). Also, by intersecting the dendrogram along the longest branches corresponds to subdividing the dataset into clusters of observations that are at a greater distance from each other within Euclidean space. In our case, cutting the dendrogram along the longest branches corresponds to a partition of the dataset in two clusters, other solutions that allow a well-spaced separation of the clusters in Euclidean space are represented by six and four clusters (Fig. D1c and D1d).

There are several quantitative clustering validation techniques that are available in the literature (Halkidi et al. 2001, Charrad et al. 2014). They can be divided into three main groups (Halkidi et al. 2001; Templ et al. 2008). The first group is based on external criteria, where the result of partitioning the dataset into clusters is evaluated by comparative analysis with an a priori known data-partition (Halkidi et al. 2001; Templ et al. 2008). The second one is based on internal criteria and tests the validity of the clustering using features that are internal to the dataset itself, as for example the distance matrix (Halkidi et al. 2001; Templ et al. 2008). The last group is based on relative criteria, which compare the final clustering scheme with other schemes. The other clustering schemes in these methods are obtained using the same algorithm but different parameter values (Halkidi et al. 2001). All the different approaches result in an index that score the number of clusters

which best represent our dataset. A collection of 30 different indices, each based on one of the above criteria, has been implemented in the R package “NbClust” (Charrad et al. 2014). Figures D2a and D2c show the application of two different cluster validation techniques: the C index (Hubert and Levin 1976), based on the comparison of distances, which returns us a number of clusters that is the more representative of the studied database the lower its value (Sheldrake and Higgins 2021) and the D-index (Lebart et al. 2000; Charrad et al. 2014), a graphic method to determine the best number of clusters and based on the clustering gain on intra-clusters inertia, which shows us a representative number of clusters at a knee in the D-index plot (Charrad et al. 2014). The number of clusters corresponding to a knee in the D-index graph is highlighted by a sharp peak in the D-index second differences plot (Fig. D2b), this peak just described correspond to the best number of clusters according to the method.

As shown in figure D2c, in the C-index plot, the best result is given for 10 clusters. According to Sheldrake and Higgins (2021), this very high number of clusters suggested by the c-index might be from over-fitting, dictated by the structure of the distance matrix. To avoid overfitting issues, Sheldrake and Higgins (2021) suggest considering the first large plateau in the C-index plot. In our case there is not a clear plateau, however, the C-index values for 5, 6 and 7 clusters do not vary much between each-others, indicating that one of these might represent a reasonable choice of cluster numbers. The D-index and the second differences D-index plots are shown in figures D2a and D2b and show a week knee around 6 clusters confirmed by a strong peak in the second differences plot. Finally, the “NbClust” package in R (Charrad et al. 2014) allows the construction of a histogram, where the x-axis shows the number of clusters, and the y-axis shows how many of the 30 indices selected that number of clusters as the best (Fig. D2d). In this case most of the indices voted for 2 clusters as the best number, and the second-best result is represented by 6. It should be noted that within the histogram, where index voting is not supervised, the c-index voted for 10 clusters as it is the number of clusters that corresponds to the lowest value of the C-index. Although the use of indices represents a more quantitative and robust method of dendrogram analysis, it is not sufficient on its own to select with certainty the true number of clusters. There is a need to understand which index is best to use and what each cluster represents within the sample. Temple et al. (2008) demonstrate how such indices are not the best approach to evaluate the cluster validity for geochemical dataset, and it should be assessed with respect to known properties of the object of investigation. In this regard, we generated a series of results in an iterative fashion, using an increasing number of clusters from 2 to 8. For each configuration we first tested the chemical validity of the clustering, and secondly ensured that each cluster corresponded to a texturally defined area of the crystal. The chemical validity was inspected by principal component analysis (PCA). For each configuration a plot of PCA1 versus PCA2 was generated, and the points were coloured according to the relative clusters, allowing us to check that each cluster is chemically well separated from the others (Fig. D1a). A visual textural analysis was conducted by plotting the analysed points on the BSE images, assigning to each point the colour of the cluster to which they belong (Fig. D1b), ensuring that for each cluster there is an area visually distinguishable and wide enough not to be the result of mixing between two areas.

As shown in Fig. D1c, using two clusters we distinguish between hourglass sectors and prism sectors. This explains why the best-number-of-cluster plot suggests two clusters as the best solution, as the chemical compositions in the hourglass and the prism are the most different from each other, which is also clearly visible from a qualitative analysis of the dendrogram. However, two clusters are not sufficient separate discrete cores and concentric zones. Six clusters are able to distinguish all the zones present in the clinopyroxenes (Fig. D1d), any number of clusters higher than six creates artificial groups that do not have any visible textural counterpart within the crystals and result into a lower quality of the chemical split in the PCA1 – PCA2 space. We conclude that in our case six is the best number of clusters.

References:

Boschetty FO, Ferguson DJ, Cortés JA, et al (2022) Insights into magma storage beneath a frequently erupting arc volcano (Villarrica, Chile) from unsupervised machine learning analysis of mineral compositions. Geochemistry, Geophysics, Geosystems 23:e2022GC010333

Caricchi L, Petrelli M, Bali E, et al (2020) A data driven approach to investigate the chemical variability of clinopyroxenes from the 2014–2015 Holuhraun–Bárdarbunga Eruption (Iceland). Frontiers in Earth Science 8:18

Charrad M, Ghazzali N, Boiteau V, Niknafs A (2014) NbClust: An R Package for Determining the Relevant Number of Clusters in a Data Set. Journal of Statistical Software 61:1–36. https://doi.org/10.18637/jss.v061.i06

Halkidi M, Batistakis Y, Vazirgiannis M (2001) On clustering validation techniques. Journal of intelligent information systems 17:107–145

Hubert LJ, Levin JR (1976) A general statistical framework for assessing categorical clustering in free recall. Psychological Bulletin 83:1072–1080. https://doi.org/10.1037/0033-2909.83.6.1072

Lebart L, Morineau A, Piron M (1995) Statistique exploratoire multidimensionnelle. Dunod Paris

Templ M, Filzmoser P, Reimann C (2008) Cluster analysis applied to regional geochemical data: Problems and possibilities. Applied Geochemistry 23:2198–2213. https://doi.org/10.1016/j.apgeochem.2008.03.004

Sheldrake T, Higgins O (2021) Classification, segmentation and correlation of zoned minerals. Computers & Geosciences 156:104876. https://doi.org/10.1016/j.cageo.2021.104876

Figure D1:

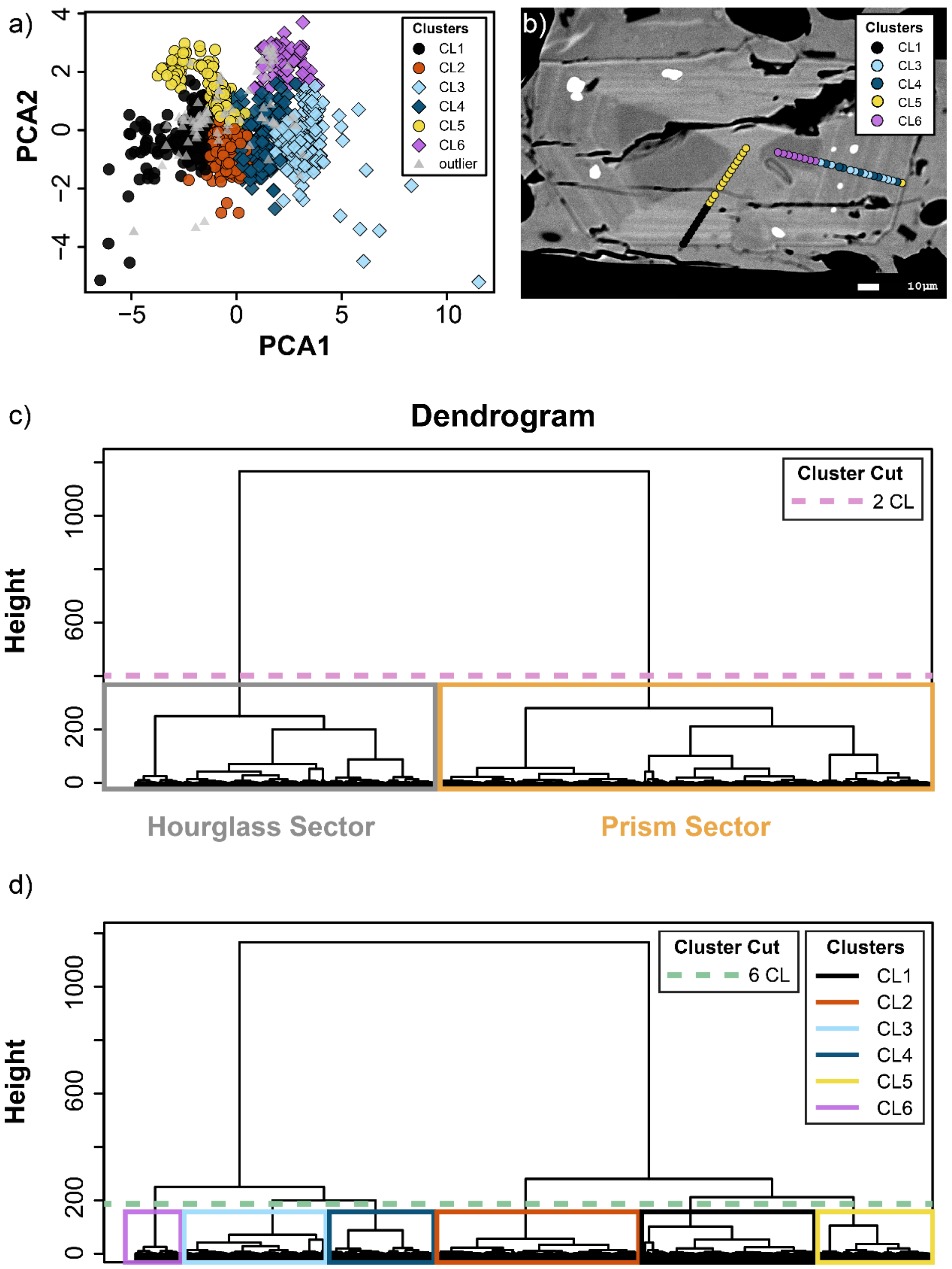

Fig. D1 – a) Plot of the first and second principal components (PCA1 and PCA2) from the principal component analysis performed on the geochemical dataset and colour contoured according to the clusters. b) BSE image on a sector zoned clinopyroxene with the analysed points coloured according to the n° of clusters. c) and d) dendrogram divided in 2 and 6 clusters respectively.

Figure D2:

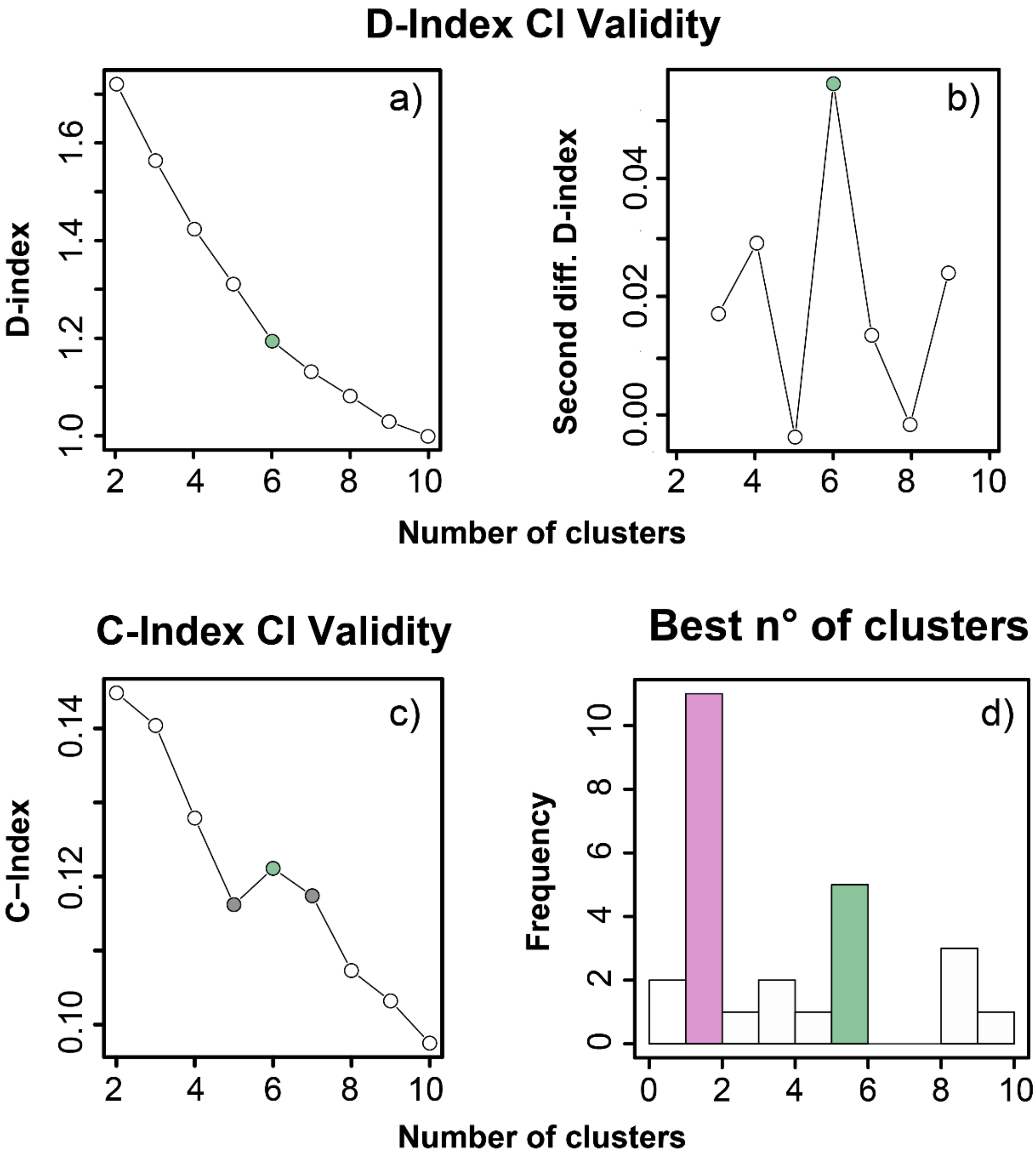

Fig. D2 – a) and b) D-index and second differences D-index values for each number of clusters, c) C-index values plotted against the number of clusters, d) Best number of clusters according to the voting method developed in the "NbClust" package in R. The pink and the grey colour highlight the 2 and 6 cluster solutions respectively.
