# Supplementary material for: The magmatic evolution of South-East Crater (Mt. Etna) during the February–April 2021 sequence of lava fountains from a mineral chemistry perspective
Source: Bull Volcanol. 2023 Apr 26;85(5):33. doi: 10.1007/s00445-023-01643-2 (PMC10133385; doi:10.1007/s00445-023-01643-2)
Supplement: Supplementary file 6 — ESM 6 (PDF 338 KB) [file 445_2023_1643_MOESM6_ESM.pdf]

Supplementary Figures

Bulletin of Volcanology (**Online Resource 6**)

Alessandro Musu1*, Rosa Anna Corsaro2, Oliver Higgins4, Corin Jorgenson1, Maurizio Petrelli3, Luca Caricchi1

1) Department of Earth Sciences, University of Geneva, rue des Maraîchers 13, 1205, Geneva, Switzerland
2) Istituto Nazionale di Geofisica e Vulcanologia, Osservatorio Etneo-Sezione di Catania, Catania, Italy.
3) Department of Physics and Geology, University of Perugia, Piazza dell'Università, 1, 06123 Perugia, Italy.
4) Geology, School of Natural Sciences, Trinity College Dublin, Dublin, Ireland
*Corresponding author (alessandro.musu@unige.ch; ORCID iD: 0000-0001-5354-5782)

Figure S1

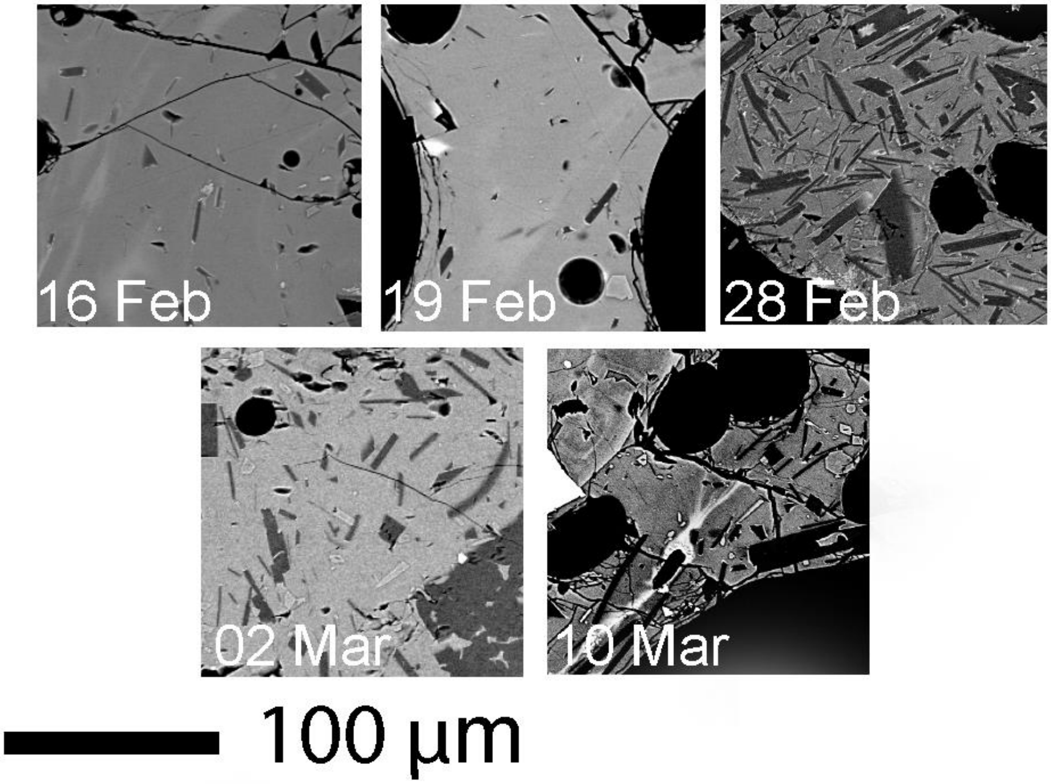

Fig. S1 – Zoomed BSE image of the groundmass of the erupted product of the 5 analyzed paroxysms. The 28 Feb event shows a higher content in microlites.

Figure S2:

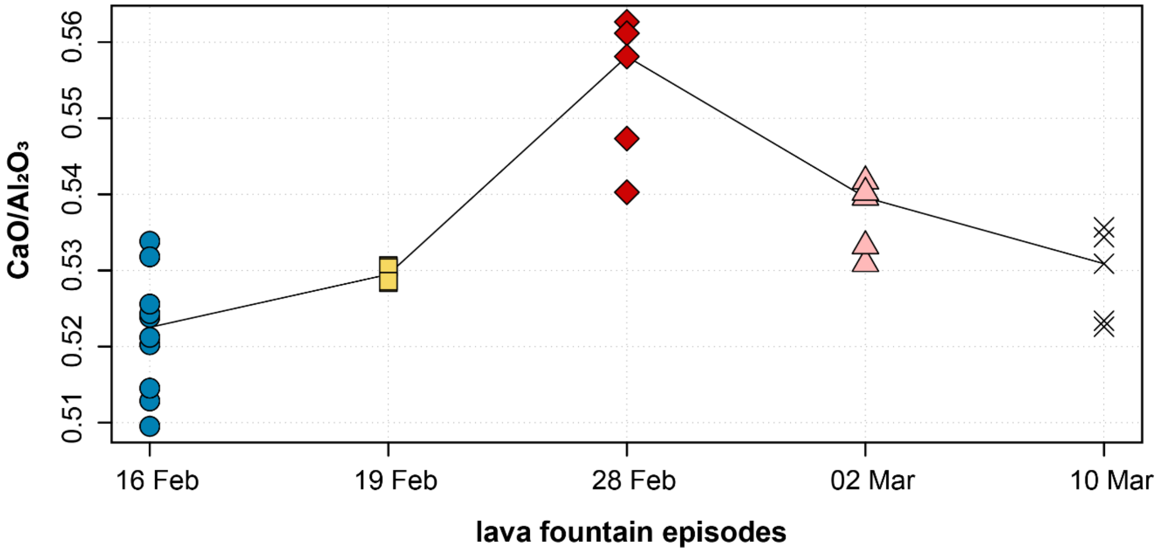

Fig. S2 – Glass compositional variation through the five analyzed eruptions, the line represents the median values.

Figure S3:

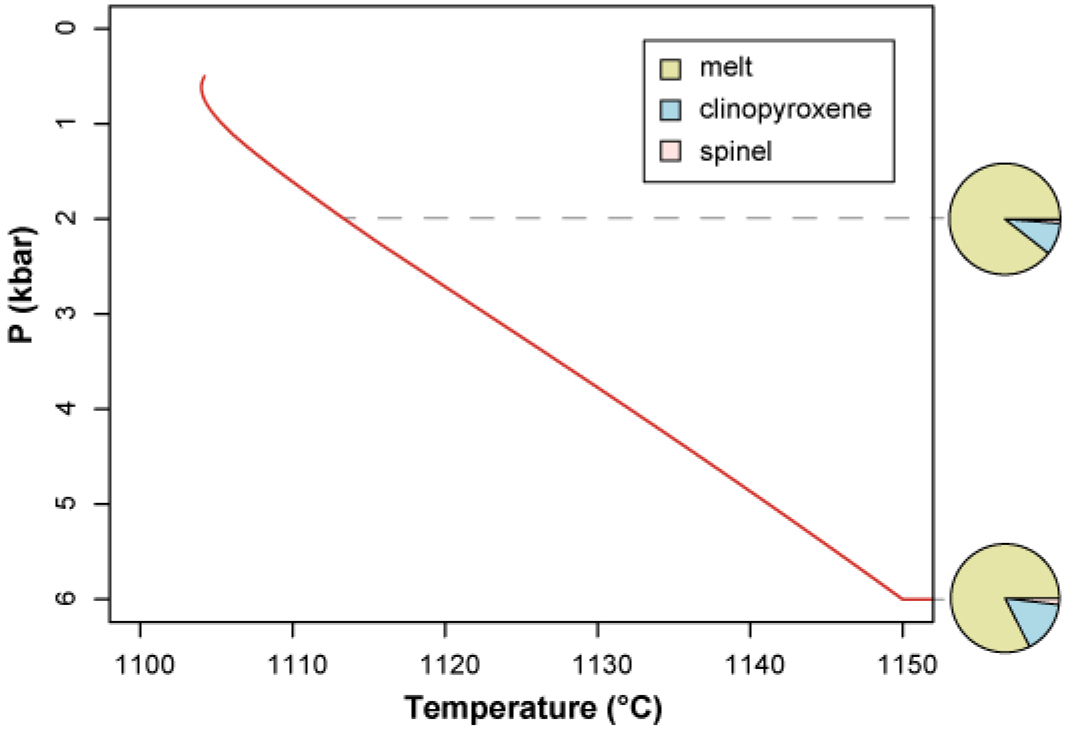

Fig. S3 – Decompression calculations performed using MELTS (Gualda et al. 2012) using the Mt. Etna compositions. Pies show mineral phase proportions at different pressure while the red line represent the melt temperature variation with pressure.

Figure S4:

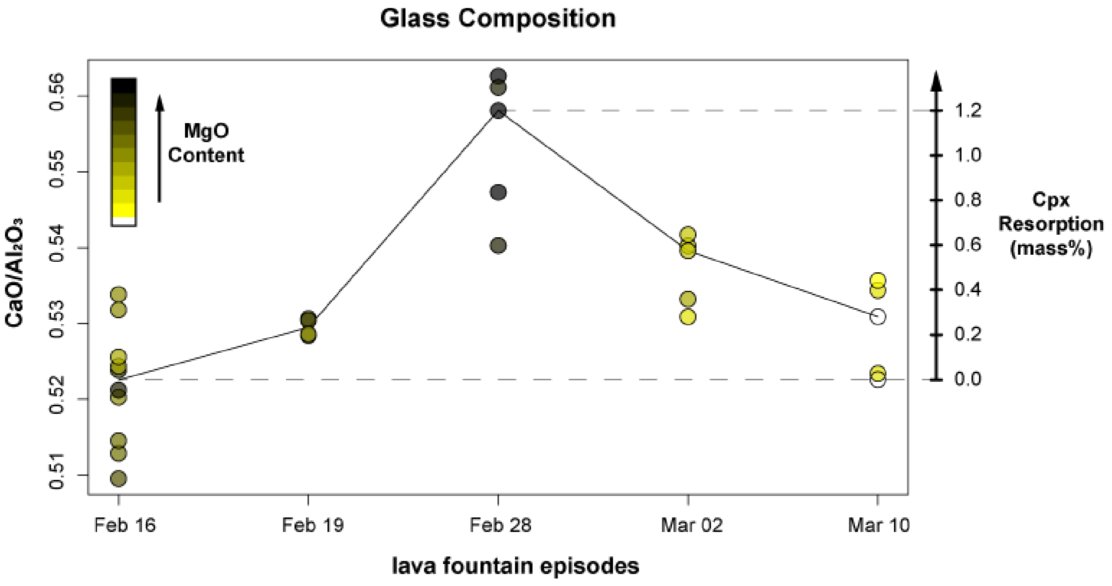

Fig. S4 – Amount of clinopyroxene (cpx) resorption necessary to explain the CaO/Al_2O_3 variation observed in the glass of the scoriae from the analyzed eruptions. The points are color-contoured for MgO content.
